# Physiological mechanism of strigolactone enhancing tolerance to low light stress in cucumber seedlings

**DOI:** 10.1186/s12870-021-03414-7

**Published:** 2022-01-13

**Authors:** Xinpeng Zhou, Zhanming Tan, Yaguang Zhou, Shirong Guo, Ting Sang, Yu Wang, Sheng Shu

**Affiliations:** 1 Key Laboratory of Southern Vegetable Crop Genetic Improvement, Ministry of Agriculture, College of Horticulture, Nanjing Agricultural University, Nanjing, 210095 People’s Republic of China; 2 College of Horticulture and Forestry Sciences, Tarim University, Xinjiang, 843300 China; 3 Institute of Horticultural Research, NingXia Academy of Agricultural and Forestry Science, YinChuan, 750002 China

**Keywords:** Antioxidant metabolism, Cucumber, Low light stress, Photosynthesis, Strigolactone, Sucrose metabolism

## Abstract

Strigolactone is a newly discovered type of plant hormone that has multiple roles in modulating plant responses to abiotic stress. Herein, we aimed to investigate the effects of exogenous GR24 (a synthetic analogue of strigolactone) on plant growth, photosynthetic characteristics, carbohydrate levels, endogenous strigolactone content and antioxidant metabolism in cucumber seedlings under low light stress. The results showed that the application of 10 μM GR24 can increase the photosynthetic efficiency and plant biomass of low light-stressed cucumber seedlings. GR24 increased the accumulation of carbohydrates and the synthesis of sucrose-related enzyme activities, enhanced antioxidant enzyme activities and antioxidant substance contents, and reduced the levels of H_2_O_2_ and MDA in cucumber seedlings under low light stress. These results indicate that exogenous GR24 might alleviate low light stress-induced growth inhibition by regulating the assimilation of carbon and antioxidants and endogenous strigolactone contents, thereby enhancing the tolerance of cucumber seedlings to low light stress.

## Introduction

Cucumber (*Cucumis sativus L.*) is an important vegetable crop. In northern China, low light is one of the main environmental factors that affects greenhouse cultivation in winter and spring. Several study results have shown that low light stress induces growth inhibition that might be related to a variety of physiological dysfunctions, including reduced photosynthetic efficiency, inhibition of biological carbon fixation, accumulation of reactive oxygen species (ROS) and membrane lipid peroxidation [1–6]. Low light stress often causes oxidative damage, which is manifested in the generation of ROS. Then, several antioxidant enzymes, such as superoxide dismutase (SOD), peroxidase (POD), and catalase (CAT), are activated in stressed plants to scavenge ROS [7, 8]. Moreover, plants acclimated to low light showed lower biomass production and higher membrane lipid peroxidation [9].

Strigolactone is a type of terpene lactone derived from carotenoids and recognized as a new type of plant hormone [10]. It plays an important role in regulating plant branch growth, stem elongation, flowering, etc. [11–15] Strigolactone plays an important role in the redistribution of nitrogen in the aboveground tissues of plants. In addition, the content of nutrient elements in the plant growth environment can affect the overall structure of the plant, and the lack of nitrogen and phosphorus will cause plant roots to become elongated, with reduced branches [16, 17]. Many studies have shown that strigolactone can also respond to abiotic stress [18–25]. In most of these studies, the changes in strigolactone were mainly related to the ABA signal regulation of stomatal closure, which indicates that strigolactone may interact with ABA signalling under stress conditions. The opening and closing of stomata are important factors that affect photosynthesis [18–20]. Ren et al. found that ABA regulates the induction of salt tolerance by SL in AM *S. cannabina* seedlings [21]. Ha et al. found that *Arabidopsis thaliana* SL-responsive max2 mutants exhibited hypersensitivity to drought and salt stress [22]. Exogenous GR24, a synthetic strigolactone analogue, can enhance osmotic stress tolerance in *Lotus japonicus* and rescue the drought-sensitive phenotype of SL-deficient mutants such as *max3* and *max4*, revealing multiple hormone-response pathways controlling adaptation to environmental stress [22, 23]. Furthermore, pretreatment with GR24 alleviated salinity stress in rapeseed (*Brassica napus L.*) and dark chilling stress in pea and Arabidopsis [24, 25]. These findings indicate that strigolactone could respond to various stresses by activating different physiological and molecular mechanisms.

However, there is little information to elucidate the mechanism of how strigolactone may alleviate the damage induced in plants under low light stress. Here, we studied the effects of exogenous GR24 on the physiological characteristics, photosynthesis and antioxidant metabolism of cucumber seedlings under low light stress to better understand the roles of strigolactone in alleviating the inhibition of growth and enhancing cucumber resistance to low light stress.

## Materials and methods

### Plant material and growth conditions

Cucumber seeds (*Cucumis sativus L.* cv Jinyou No. 4) were purchased from Tianjin Kerun Cucumber Research Institute (Tianjin, China). The seeds were soaked in warm water at 55 °C for 3 ~ 4 h and then wrapped with wet gauze. The seeds were placed in a petri dish for germination at 29 ± 1 °C. The germinated seeds were sown in a growth substrate (pH = 6.3 ± 0.2, EC = 2.1 ± 0.2 mS·cm^− 1^), which consisted of vermiculite and perlite (3:1, v/v), in plastic containers. The growth environment conditions were maintained as follows: 28/18 °C (day/night) temperature, a mean relative humidity from 70 ~ 80%, and 14/10 h (day/night) photoperiods with active photosynthetic photon flux density (PPFD) maintained at 400 ~ 800 μmol·m^− 2^·s^− 1^. After the second true leaves were fully expanded, cucumber seedlings were selected for transfer to plastic tanks. GR24 (strigolactone analogue) was purchased from Nanjing Wobo Biological Technology Co., Ltd. (Nanjing, China).

### Low light and GR24 treatments

When the seedlings reached the three-true-leaf stage, the experimental plots included four treatments:

1. Cont: Cucumber seedlings were planted in a growth substrate with half-strength Hoagland nutrient solution and sprayed with 0.02% acetone on the leaves under PPFD maintained at 500 μmol·m^− 2^·s^− 1^;
2. LL: Cucumber seedlings were planted in a growth substrate with half-strength Hoagland nutrient solution, and 0.02% acetone was sprayed on the leaves under a PPFD of 60 μmol·m^− 2^·s^− 1^;
3. GR24: Cucumber seedlings were planted in a growth substrate with half-strength Hoagland nutrient solution, and the leaves were sprayed with 10 μM GR24 solution dissolved in 0.02% acetone under a PPFD maintained at 500 μmol·m^− 2^·s^− 1^;
4. LL + GR24: Cucumber seedlings were planted in a growth substrate with half-strength Hoagland nutrient solution and sprayed with 10 μM GR24 solution, which was dissolved in 0.02% acetone on the leaves under PPFD maintained at 60 μmol·m^− 2^·s^− 1^.

GR24 was dissolved in 0.02% acetone according to the methods of Alessandro Luisi et al. [26]. All groups were established at day 0 and irrigated with half-strength Hoagland’s nutrient solution (pH 6.5 ± 0.1, EC 2.0–2.2 mS·cm^− 1^) simultaneously. Each group was sprayed with the appropriate solution once a day for 7 days. All leaves were sprayed with GR24 solution and 0.02% acetone every day at 8.00 a.m. Each treatment was arranged in a completely randomized block design with three replicates containing 36 plants for each replicate. The GR24 concentration and the active photosynthetic photon flux density were selected on the basis of our preliminary test. After 7 days of treatment, the third fully expanded leaf was taken from the top of each plant for further biochemical analysis.

### Growth parameters

The outline of the third true leaf under the growth point was traced on A4 paper (21 cm × 29.7 cm) and cut out, and then an electronic balance was used to weigh the mass; the ratio of n to the mass of the complete A4 paper was calculated, and then the leaf area was calculated as n × A4 paper area (21 × 29.7 cm^2^). The fresh weight was weighed with an electric balance after washing with sterile distilled water. For measuring dry weight (DW), seedlings were put in paper bags and labelled and dried in an oven for 15 min at 105 °C and then for 72 h at 70 °C.

### Chlorophyll content and gas exchange parameters

According to the methods of Shu et al. [4], to determine the chlorophyll content in cucumber seedlings subjected to low light stress and GR24 treatment, 0.1 g leaves were homogenized in 80% acetone and then centrifuged; the supernatant was collected, and the chlorophyll contents were analysed with a UV-1800 spectrophotometer. Gas exchange parameters such as the net photosynthetic rate (Pn), stomatal conductance (Gs), transpiration rate (Tr), and intercellular CO_2_ concentration (Ci) were monitored using a portable photosynthesis system (LI-6400XT, LI-COR Inc., USA). The leaf chamber temperature was 25 ± 1 °C, light intensity was 1000 μmol m^− 2^ · s^− 1^, CO_2_ concentration was 400 ± 10 μmol·mol^− 1^, and relative humidity was maintained at 60 ~ 70%.

### Chlorophyll fluorescence imaging

According to the methods of Yuan et al. [27] and Wu et al. [28], chlorophyll fluorescence imaging of cucumber leaves was performed using a modulated fluorescence imager (Imaging-PAM, Walz, Germany), and plants were fully adapted in the dark before measurement. The chlorophyll fluorescence parameters were calculated in accordance with Wu et al. [29]. The chlorophyll fluorescence parameters were calculated as follows: maximum photochemical efficiency of PSII (Fv/Fm): Fv/Fm = (Fm − Fo)Fm; effective photochemical quantum yield of PSII: ФPSII = (Fm′ − Fs)/Fm′: regulatory energy dissipation quantum yield of PSII: ФNPQ = 1 − ФPSII− 1/[NPQ + 1 + qL(Fm/Fo − 1)]; nonregulatory energy dissipation quantum yield of PSII: ФNO = 1/[NPQ + 1 + qL(Fm/Fo − 1)]; nonphotochemical quenching: NPQ = (Fm − Fm′)/Fm′; photochemical quenching coefficient: qP = (Fm − Fs)/(Fm′ - Fo’). Here, Fm, is the maximum fluorescence yield; Fo, is the minimum fluorescence yield; Fs, is the steady-state fluorescence yield; Fm′, is the fluorescence maximum in the light; qL, is photoinhibitory quenching.

### Carbohydrate content

The contents of total soluble sugar and sucrose were evaluated following the methods of Shen et al. [30]. After drying the plant sample, 0.15 g dry sample was dissolved in 80% ethanol in a water bath at 80 °C for 30 min. Extraction was performed 3 times, and the resulting supernatant was used for the determination of soluble total sugar and sucrose.

### Enzyme activities of sucrose metabolism

The activities of sucrose synthase (SS) and sucrose phosphate synthase (SPS) were determined using reagent detection kits (Beijing Solarbio Science & Technology Co., Ltd., China). Samples (0.1 g) were ground in 1 mL extract, and the homogenate was centrifuged at 8000×g for 10 min at 4 °C. The enzyme activities were estimated with the extracted supernatants. Following the instructions of the kit, the activities of SS and SPS were recorded at 480 nm with a spectrometer, and SS and SPS were expressed in μg·min^− 1^·g^− 1^ FW.

### Endogenous strigolactone content

The content of endogenous strigolactone was determined using a Plant SLs ELISA Kit (Shanghai Renjie Biological Technology Co., Ltd., China) following the company’s instructions. Briefly, after the sample was fully ground in liquid nitrogen, the mixture was added to 9-fold the sample volume of the extract followed by centrifugation at 8000×g for 30 min at 4 °C. For the enzyme-linked immunosorbent assay, the extracted supernatants and strigolactone antibody were kept in the microplate and incubated and washed thoroughly. Colour development was determined with the substrate tetramethylbenzidine (TMB). The strigolactone content was assessed by an Infinite F50 microplate reader, and based on the standard curve, the concentrations were calculated with the absorbance value recorded at 450 nm.

### H_2_O_2_ and MDA contents

According to the methods of Zhang et al. [30] and Shu et al. [31]. For the estimation of H_2_O_2_, a 0.3 g sample was homogenized in 3 mL of 0.1% trichloroacetic acid (TCA) (w/v) followed by centrifugation at 12000×g for 15 min at 4 °C. Then, 0.5 mL of 0.1 M potassium phosphate buffer (pH 7.8) and potassium iodine (1 mL 1 M KI) were incorporated into the supernatant (0.5 mL) and placed in the dark for 1 h. The mixture was used to determine H_2_O_2_. The content of H_2_O_2_ was expressed as μmol·g^− 1^ FW. After grinding the sample, a 1 g sample was dissolved in TCA in a water bath at 95 °C for 30 min and centrifuged at 1500 g for 10 min. The absorbance was recorded at 532 nm and 600 nm. MDA content was expressed by nmol·g^− 1^ FW.

### Antioxidant enzyme activity and antioxidant content

Fresh samples (0.1 g) were ground in 1.5 mL of 50 mM precooled phosphate buffer (pH 7.8), and the homogenate was centrifuged at 12000×g for 20 min at 4 °C. The activities of antioxidant enzymes were estimated with the extracted supernatants. The activities of ascorbate peroxidase (APX), glutathione reductase (GR), dehydroascorbate reductase (DHAR) and monodehydroascorbate reductase (MDHAR) were estimated by the methods of Shu et al. [32]. According to the methods of Jahan et al. [33], the ascorbate (AsA) and oxidized glutathione (GSSG) contents were recorded at 265 nm and 412 nm with a spectrometer, respectively. Oxidized ascorbate (DAsA) was estimated by subtracting the reduced AsA from total AsA. The content of reduced glutathione (GSH) was calculated by the difference in GSSG from the total GSH.

### Real-time quantitative PCR analysis (qRT–PCR)

Total RNA was extracted from leaves using a total RNA extraction kit (RNA simple Total RNA Kit, Tiangen Biotech Co., Ltd., China). The RNA concentration was measured by a spectrophotometer, rendering the final concentration 100 ng·μL^− 1^. Total RNA was reverse transcribed into single-stranded cDNA using the PrimeScriptTM First-Strand cDNA Synthesis Kit (Takara Bio Inc., Japan) for qRT–PCR analysis. With reverse transcription cDNA as the template, the gene fragment was amplified. Real-time fluorescent quantitative PCR was carried out using ChamQ SYBR qPCR Master Mix (Vazyme Biotech Co., Ltd., China) to form a 20 μL reaction with cDNA. Gene-specific primers were designed from the NCBI and the Cucumber Genome Database (cucumber.genomics.org.cn), and actin served as an internal control (Table 1). Each sample was repeated three times. Amplification was performed by a StepOneTM real-time quantitative PCR system (Applied Biosystems, Singapore).

**Table 1:** Primers used for qRT–PCR analysis and their target genes

Gene name	Direction	Primer sequence
*RBOH*	F	5′ AAGGTTGCTGTTTATCC 3′
	R	5′ AATGGTCTTGAGTTGGG 3′
*SS*	F	5′ CGGTTACTTCGCACAAGATAATG3’
	R	5′ CATAACCAGATCACCAGACTCC 3′
*SPS*	F	5’GAGAGGATTCCGGTGCAATATC 3′
	R	5′ CAAGAAGACAGGCACTAAGGTAC 3′
*APX*	F	5′ TGCTTTCATCACCATCAA 3′
	R	5′ TGTTATGTTCTTGTCTTCCT 3′
*GR*	F	5′ CATGGTGCTGTGAAG 3′
	R	5′ TCTTACACCTGTGGCTCTAATG 3′
*DHAR*	F	5’GGCAAAGTTCCCGTTGTTAAAT 3′
	R	5′ AAGAGAAGTACCCAAATCCACC 3′
*MDHAR*	F	5′ GCGAGGGAGTTTGTGAAATTG 3’
	R	5′ GTATCTCTTCCCTGAGTCTCCT 3’
*MAX*_*1*_	F	5′ CCCATACTCCAAGAGCCATTTA 3’
	R	5′ AAGCCTACCACTCAACTTCTG 3’
*MAX*_*2*_	F	5′ ACCAGGAAAAACCATAGACAGAA3’
	R	5′ TGACGAGCAGAGGAACGC 3’
*MAX*_*3*_	F	5′ TGCCACCGAAGAGGTTATTG 3’
	R	5′ TGGCTAGTTGGTAGAAGCATATC 3’
*MAX*_*4*_	F	5′ GGAGATCGACACCTCGAATAAT 3’
	R	5′ CACTCAGCCAAGAGGGTAAA 3’
*Actin*	F	5′ CAGGAATCCACGAAACTACT 3’
	R	5′ AGACCCTCCAATCCAAACAC 3’

Note: *RBOH* respiratory burst oxidase homologue, *SS* sucrose synthase, *SPS* sucrose phosphate synthase, *APX* ascorbate peroxidase, *GR* glutathione reductase, *DHAR* dehydroascorbate reductase, *MDHAR* monnodehydroascorbate, *MAX*_*1*_*(CYP711A1)* more axillary growth1(P450 monooxygenase), *MAX*_*2*_ more axillary growth2(F-box protein of a SCF complex), *MAX*_*3*_*(CCD*_*7*_*)* more axillary growth3(carotenoid cleavage dioxygenase7), *MAX*_*4*_*(CCD*_*8*_*)* more axillary growth4(carotenoid cleavage dioxygenase8); Actin, housekeeping gene

### Data analysis

The whole experiment was performed in triplicate. All the data were analysed statistically using SPSS 20.0 (SPSS Inc., Chicago, IL, USA) software according to Duncan’s multiple comparison method (*P <* 0.05).

## Results

### Effects of GR24 on growth performance under low light stress

**Fig. 1:**
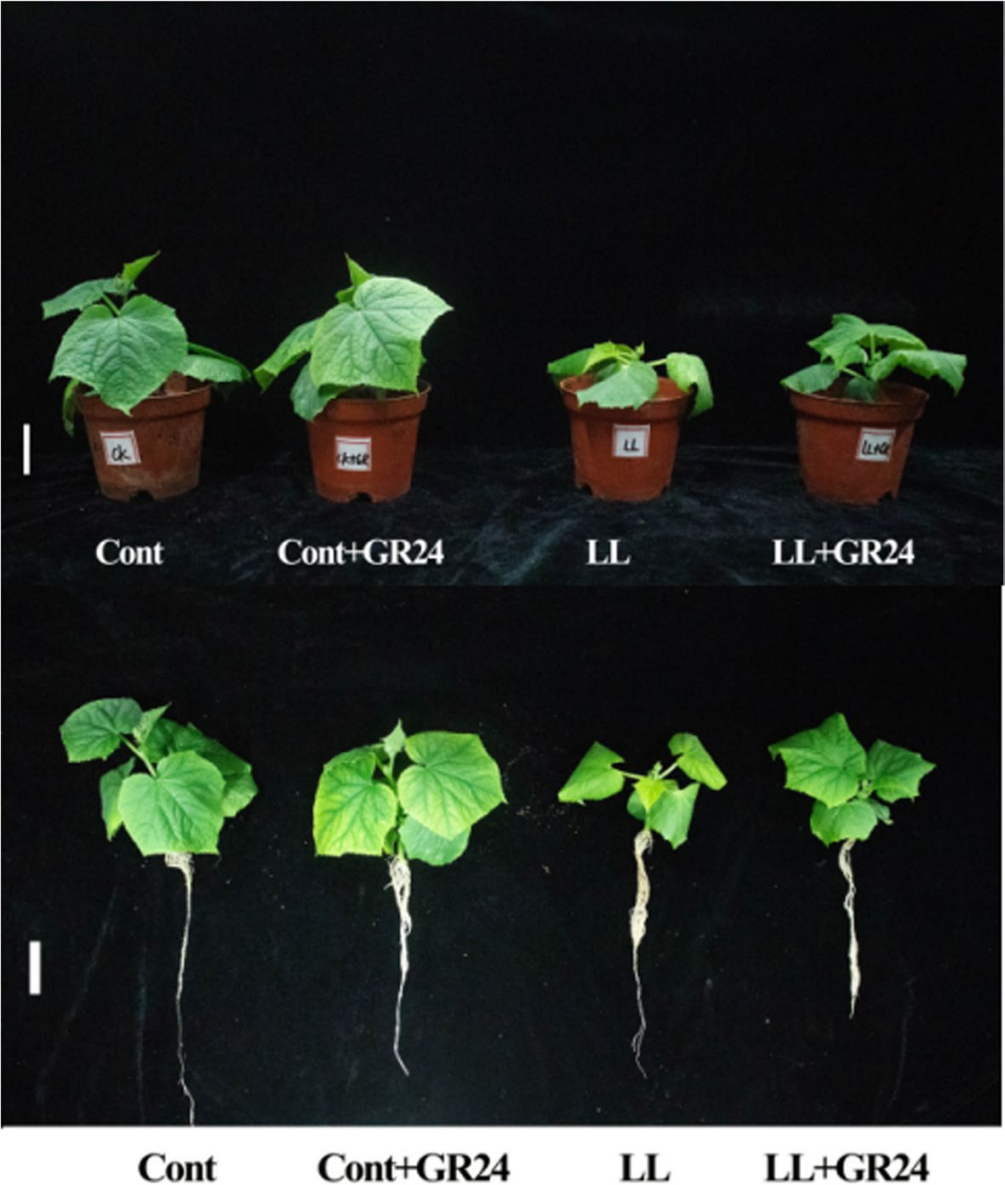
Visual assessment of cucumber seedlings under low light and GR24 treatment conditions. Note: Photographs of the cucumber seedlings were taken at the end of different treatments. The growth of cucumber seedlings was significantly inhibited under different treatments for 7 d. Cont, 0 μM GR24 + 500 μmolm^− 2^ s^− 1^ PPFD; Cont+GR24, 10 μM GR24 + 500 μmolm^− 2^ s^− 1^ PPFD; LL, 0 μM GR24 + 60 μmolm^− 2^ s^− 1^ PPFD; LL + GR24, 0 μM GR24 + 60 μmolm^− 2^ s^− 1^ PPFD

Low light stress significantly inhibited the growth performance and biomass of cucumber seedlings (Fig. 1). As shown in Table 2, compared with the control, the plant height, stem diameter, shoot dry weight and shoot fresh weight decreased by 25.1, 36.3, 64.2 and 49.1% under low light, respectively. Low light stress also significantly decreased leaf length, leaf width, leaf area, root dry weight and root fresh weight by 12.9, 24.6, 34.51, 57.1 and 58.7%, respectively. However, exogenous application of GR24 significantly improved the adverse effects of low light stress on the inhibition of plant growth. Under the control conditions, GR24 alone significantly increased leaf size but had no significant effects on other growth parameters.

**Table 2:** Effects of exogenous GR24 treatments on the growth parameters of cucumber seedlings under low light stress

Treatments	Fresh weight/(g·plant^− 1^)			Dry weight/(g·plant^− 1^)		
	Shoot	Root	Total	Shoot	Root	Total
Cont	7.59 ± 1.70a	3.44 ± 0.51a	11.03 ± 1.32a	1.23 ± 0.22a	0.14 ± 0.02a	1.37 ± 0.20a
Cont+ GR24	7.54 ± 0.90a	3.46 ± 0.83a	11.00 ± 0.88a	1.20 ± 0.09a	0.16 ± 0.02a	1.36 ± 0.08a
LL	3.86 ± 0.78c	1.42 ± 0.27c	5.28 ± 0.64c	0.44 ± 0.07c	0.06 ± 0.02c	0.50 ± 0.06c
LL + GR24	4.83 ± 0.57b	1.82 ± 0.41b	6.65 ± 0.52b	0.67 ± 0.03b	0.08 ± 0.03b	0.75 ± 0.03b
Treatments	Leaf length (cm)	Leaf width (cm)	Leaf area (cm^2^)	Plant height (cm)		Stem diameter (mm)
Cont	12.72 ± 0.22c	10.06 ± 0.29b	94.78 ± 3.06b	25.54 ± 1.71a		4.79 ± 0.38a
Cont+ GR24	14.45 ± 0.83a	11.73 ± 1.12a	112.49 ± 9.12a	25.57 ± 1.35a		4.78 ± 0.37a
LL	11.08 ± 0.38d	7.59 ± 0.24d	62.07 ± 1.68d	19.13 ± 1.87b		3.05 ± 0.04c
LL + GR24	13.37 ± 0.42b	8.41 ± 0.53c	68.92 ± 4.76c	19.66 ± 1.86b		3.56 ± 0.03b

Note: Each value represents the means ± SEs. Different letters in the same column indicate significant differences at *P* < 0.05 according to Duncan’s multiple range tests. Cont, 0 μM GR24 + 500 μmolm^−2^ s^−1^ PPFD; Cont+GR24, 10 μM GR24 + 500 μmolm^− 2^ s^− 1^ PPFD; LL, 0 μM GR24 + 60 μmolm^− 2^ s^− 1^ PPFD; LL + GR24, 10 μM GR24 + 60 μmolm^− 2^ s^− 1^ PPFD

### Effects of GR24 on the photosynthetic pigment content and gas exchange parameters under low light stress

**Fig. 2:**
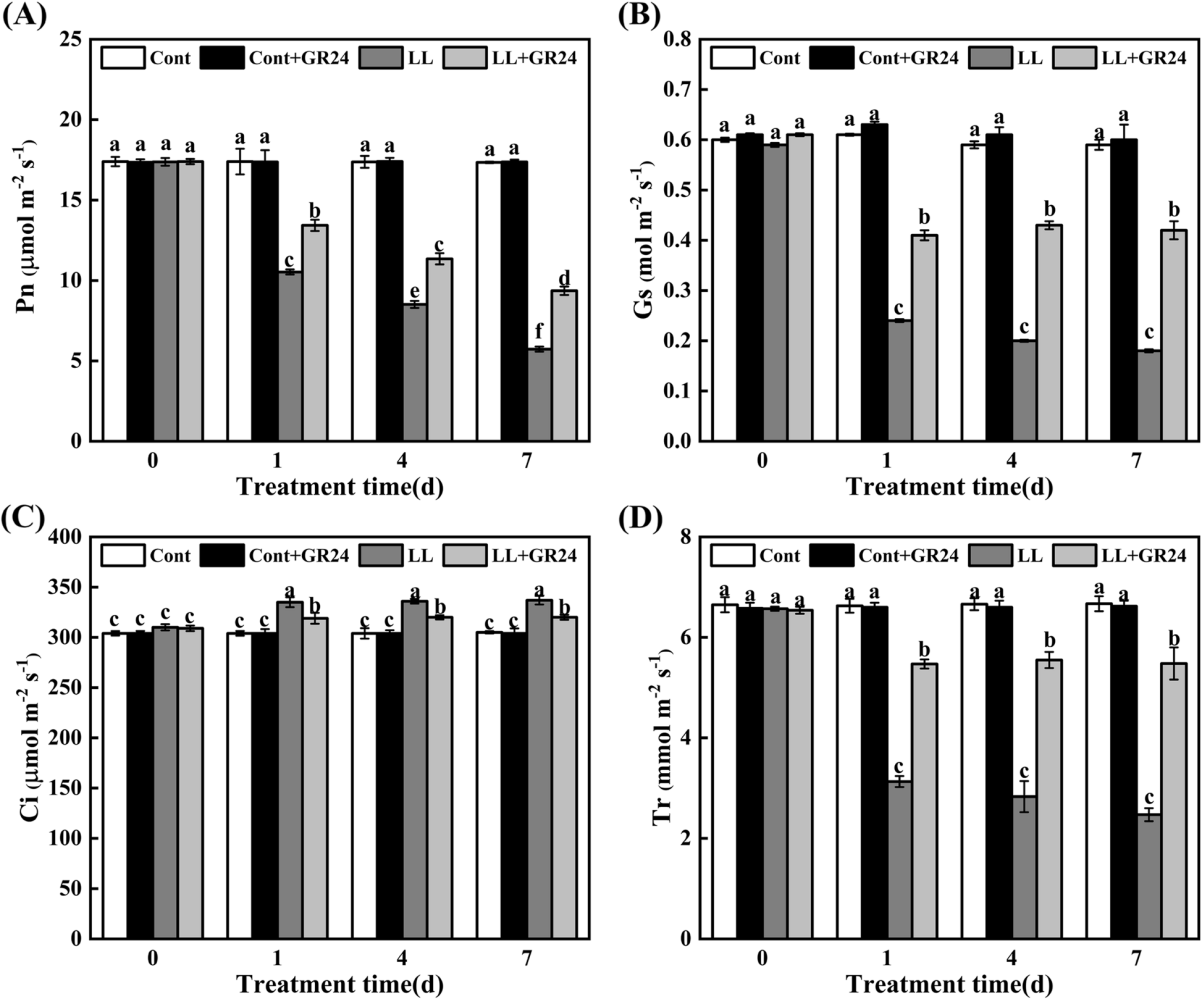
Effects of exogenous GR24 on the gas exchange parameters Pn (**A**), Gs (**B)**, Ci (**C**) and Tr (**D**) in cucumber seedlings under low light stress. Note: The respective parameters were measured at 0, 1, 4, and 7 days after the start of low light stress and/or 10 μM GR24 treatments. Each histogram represents the mean value of three independent experiments, and the vertical bars indicate SEs (*n* = 3). Different letters indicate significant differences at *P* < 0.05, according to Duncan’s multiple range tests. Cont, 0 μM GR24 + 500 μmolm^− 2^ s^− 1^ PPFD; Cont+GR24, 10 μM GR24 + 500 μmolm^− 2^ s^− 1^ PPFD; LL, 0 μM GR24 + 60 μmolm^− 2^ s^− 1^ PPFD; LL + GR24, 10 μM GR24 + 60 μmolm^− 2^ s^− 1^ PPFD. Here, Pn, net photosynthetic rate; Gs, stomatal conductance; Ci, intercellular CO_2_ concentration; Tr, transpiration rate

As shown in Table 3, compared with the control, low light stress increased chlorophyll *b* (Chl *b*) by 22.3% but decreased the chlorophyll *a* (Chl *a*), total chlorophyll content (Chl *a + b*) and Chl *a/b* of cucumber seedling leaves by 30.00, 9.8 and 42.8%, respectively. Exogenous GR24 induced a significant increase in the levels of Chl *a*, Chl *a + b* and Chl *a/b*. There was no significant difference in Chl content between the control leaves and GR24-treated leaves. With increasing treatment time, the net photosynthetic rate (Pn), stomatal conductance (Gs) and transpiration rate (Tr) of cucumber leaves significantly decreased under low light stress. On the seventh day, these values decreased to 33.03, 30.51, and 37.03% of the control levels, but the intercellular carbon dioxide concentration (Ci) increased by 10.49%. However, exogenous GR24 alleviated the negative effects caused by low light stress, thus increasing the values of Pn, Gs and Tr by 63.35, 133.33 and 121.86%, respectively. No significant differences were observed in photosynthetic parameters between the control and GR24-treated seedlings (Fig. 2).

**Table 3:** Effects of exogenous GR24 treatments on chlorophyll contents in leaves of cucumber seedlings under low light stress

Treatments	Chl *a* (mg·g^−1^)	Chl *b* (mg·g^− 1^)	Total Chl (mg·g^− 1^)	Chl *a/b*
Cont	1.50 ± 0.02b	0.94 ± 0.02b	2.44 ± 0.02a	1.59 ± 0.04b
Cont+ GR24	1.53 ± 0.03b	0.90 ± 0.01bc	2.43 ± 0.02a	1.70 ± 0.05a
LL	1.05 ± 0.03c	1.15 ± 0.01a	2.20 ± 0.04b	0.91 ± 0.02c
LL + GR24	1.59 ± 0.01a	0.88 ± 0.03c	2.47 ± 0.04a	1.80 ± 0.06a

Note: The growth of cucumber seedlings was significantly inhibited under different treatments for 7 d. The values are the means ± SEs (*n* = 3). The different letters indicate significant differences at *P* < 0.05 according to Duncan’s multiple range test. Cont, 0 μM GR24 + 500μmolm^− 2^s^−1^PPFD; Cont+GR24, 10 μM GR24 + 500μmolm^− 2^ s^− 1^ PPFD; LL, 0 μM GR24 + 60μmolm^− 2^ s^− 1^ PPFD; LL + GR24, 10 μM GR24 + 60μmolm^− 2^ s^− 1^ PPFD

### Effects of GR24 on the chlorophyll fluorescence parameters of cucumber leaves under low light stress

**Fig. 3:**
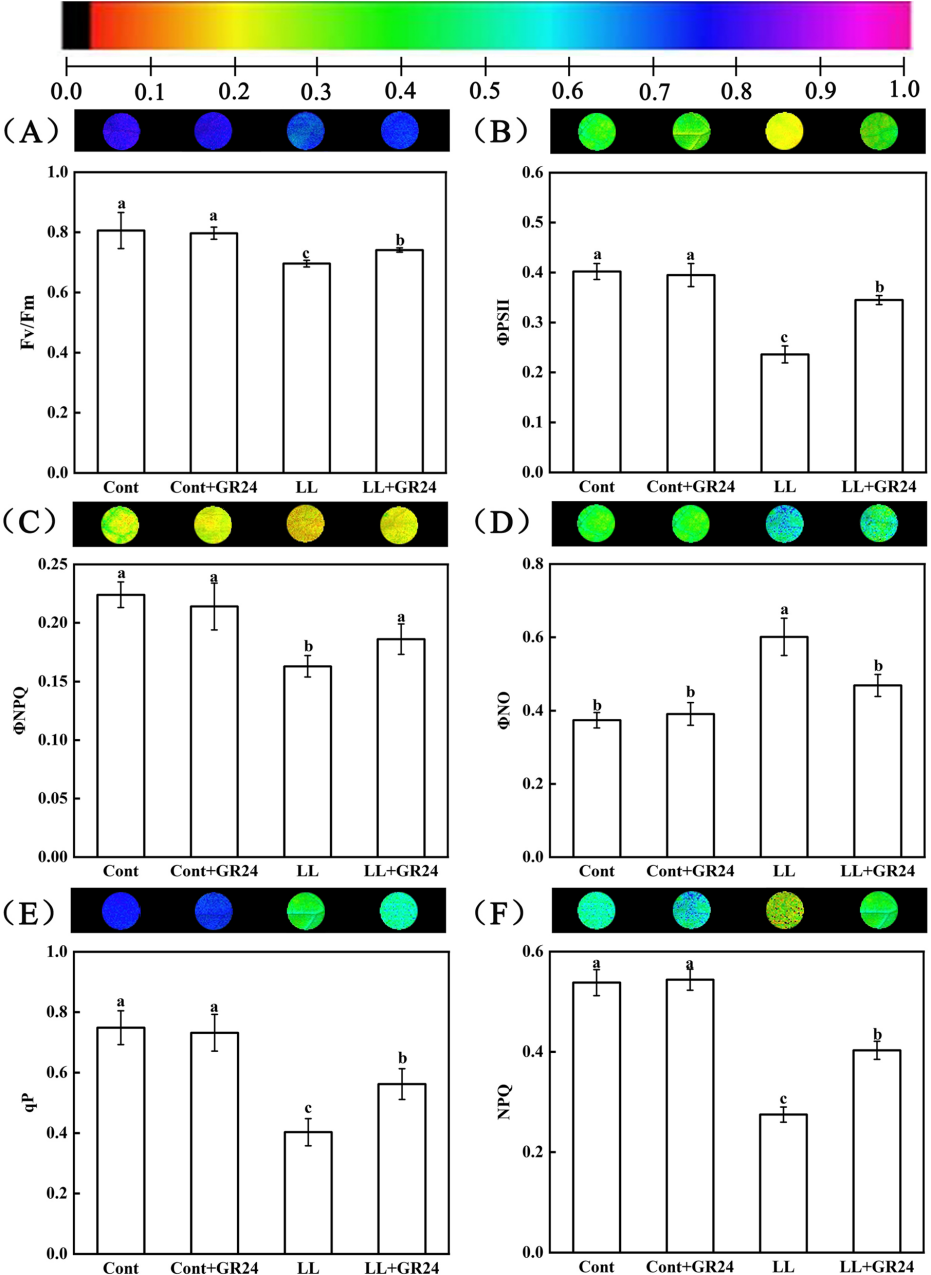
Effects of exogenous GR24 on chlorophyll fluorescence in cucumber seedlings under low light stress. Note: Here, Fv/Fm, the maximum quantum yield of PSII; ФPSII, actual photochemical efficiency of PSII; ФNPQ, modulated heat dissipation of PSII; ФNO, nonmodulated heat dissipation of PSII; qP, photochemical quenching coefficient; NPQ, nonphotochemical quenching coefficient. Each image in the same column represents the same leaf. The bars represent the standard errors. The colour scale at the bottom indicates values from 0 (black) to 1 (pink)

As shown in Fig. 3, under normal growth conditions, GR24 had no significant effects on the maximum quantum yield of PSII (Fv/Fm), actual photochemical efficiency of PSII (ФPSII), modulated heat dissipation of PSII, (ФNPQ), nonmodulated heat dissipation of PSII (ФNO), photochemical quenching coefficient (qP), or nonphotochemical quenching coefficient (NPQ). However, compared with the control, low light stress reduced the values of Fv/Fm, ФPSII, ФNPQ, qP and NPQ by 13.65, 41.29, 27.23, 46.19 and 48.88%, respectively, but significantly increased ФNO. However, the application of GR24 under low light increased the values of Fv/Fm, ФPSII, ФNPQ, qP and NPQ by 6.47, 46.19, 14.11, 39.45 and 46.55%, respectively, but reduced the value of ФNO by 21.96% under low light stress.

### Effects of GR24 on the carbohydrate level and sucrose metabolism of cucumber seedlings under low light stress

**Fig. 4:**
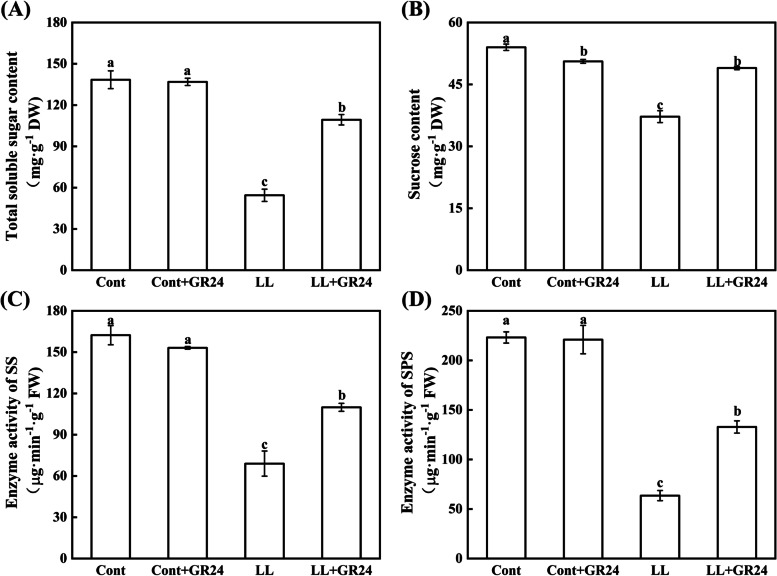
Effects of exogenous GR24 on the content of soluble total sugar (**A**) and sucrose (**B**) and enzyme activity related to sucrose metabolism in cucumber seedlings under low light stress. Note: The respective parameters were measured at 7 days after the start of low light stress and/or 10 μM GR24 treatments. Each histogram represents the mean value of three independent experiments, and the vertical bars indicate SEs (*n* = 3). Different letters indicate significant differences at *P* < 0.05, according to Duncan’s multiple range tests. Here, SS, sucrose synthase; SPS, sucrose phosphate synthase. Cont, 0 μM GR24 + 500 μmolm^− 2^ s^− 1^ PPFD; Cont+GR24, 10 μM GR24 + 500 μmolm^− 2^ s^− 1^ PPFD; LL, 0 μM GR24 + 60 μmolm^− 2^ s^− 1^ PPFD; LL + GR24, 10 μM GR24 + 60 μmolm^− 2^ s^− 1^ PPFD

**Fig. 5:**
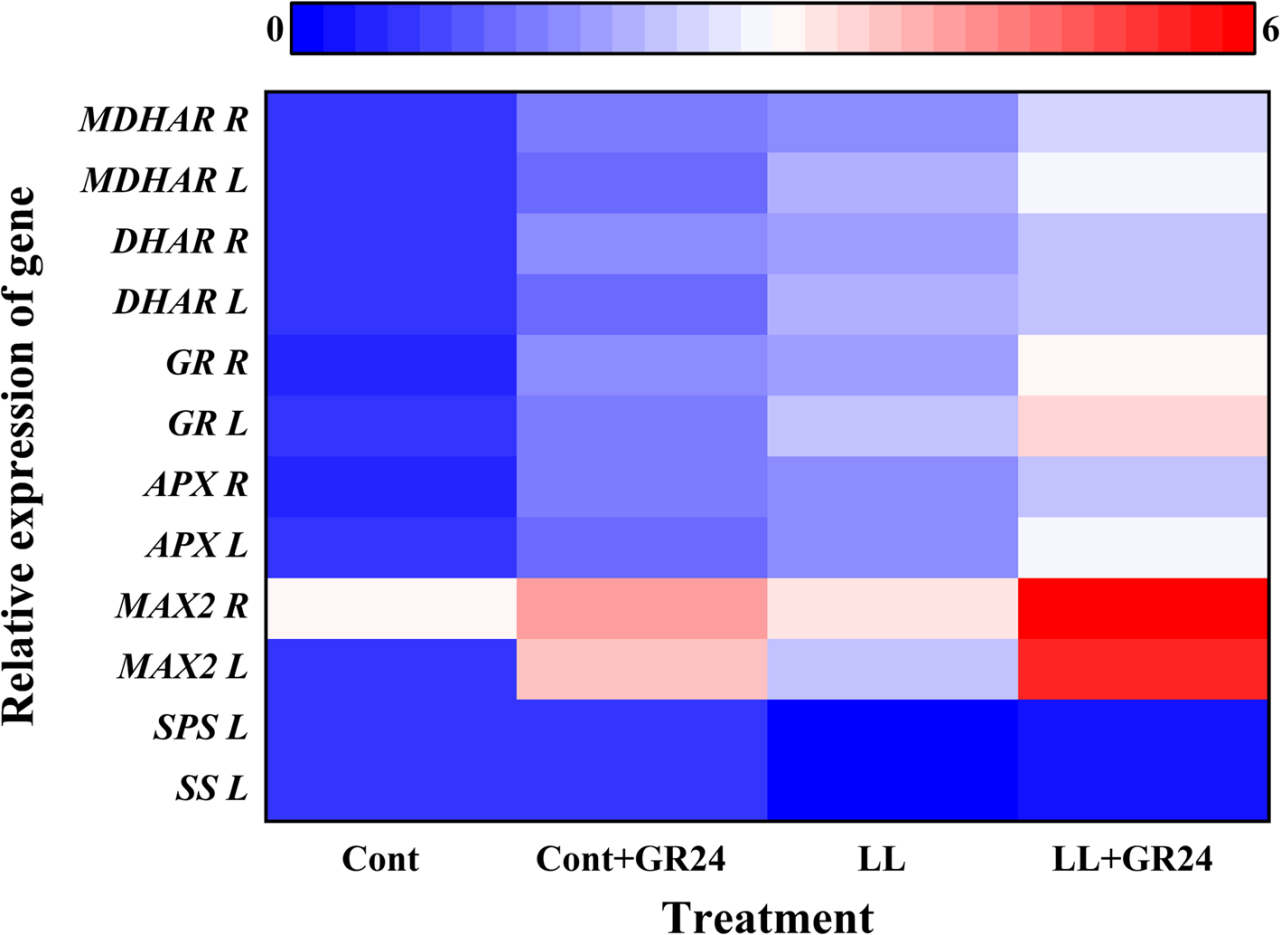
Heatmap representing the relative transcript abundance of differentially expressed antioxidant enzyme-encoding genes, strigolactone Signaling genes, and sucrose metabolism enzyme genes in the leaves and roots of cucumber seedlings under low light stress with or without GR24 treatment. Note: The gene expression intensity is represented with a colour gradient from blue (low) to red (high). The respective parameters were measured at 7 days after the start of low light stress and/or 10 μM GR24 treatments. Cont, 0 μM GR24 + 500 μmolm^− 2^ s^− 1^ PPFD; Cont+GR24, 10 μM GR24 + 500 μmolm^− 2^ s^− 1^ PPFD; LL, 0 μM GR24 + 60 μmolm^− 2^ s^− 1^ PPFD; LL + GR24, 10 μM GR24 + 60 μmolm^− 2^ s^− 1^ PPFD. Here, *MDHAR R*, monnodehydroascorbate in roots; *MDHAR L*, monnodehydroascorbate in leaves; *DHAR R*, dehydroascorbate reductase in roots; *DHAR L*, dehydroascorbate reductase in leaves; *GR R*, glutathione reductase in roots; *GR L*, glutathione reductase in leaves; *APX R*, ascorbate peroxidase in roots; *APX L*, ascorbate peroxidase in leaves; *MAX*_*2*_ *R*, more axillary growth 2 in roots; *MAX*_*2*_ *L*, more axillary growth 2 in leaves; *SPS L*, sucrose phosphate synthase in leaves; *SS L*, sucrose synthase in leaves

As shown in Fig. 4A and B, compared with the control, low light stress caused a significant decrease in the soluble sugar and sucrose contents of cucumber leaves. However, compared with low light stress alone, GR24 supplementation under low light stress significantly increased the levels of soluble sugar and sucrose by 100.83 and 31.67%, respectively. To gain a better understanding of how GR24 enhances sucrose metabolism, we quantified sucrose synthase enzyme and sucrose phosphate synthase enzyme activity in cucumber leaves (Fig. 4C and D) and *SS* (sucrose synthase) and *SPS* (sucrose phosphate synthase) gene expression (Fig. 5). Compared with the control, low light stress reduced the activities of sucrose synthase and sucrose phosphate synthase by 57.50 and 71.54%, respectively, and the corresponding SS and SPS gene expression levels were 0.42- and 0.28-fold higher than those of the control, respectively. Interestingly, supplementing GR24 under low light stress increased the activities of sucrose synthase and sucrose phosphate synthase by 59.28 and 109.12%, respectively, compared with those under only low light stress, while the expression levels of the corresponding coding genes *SS* and *SPS* were 1.59- and 2.09-fold higher than those under low light stress.

### Effects of GR24 on the biosynthesis and signal transduction of strigolactone under low light stress

**Fig. 6:**
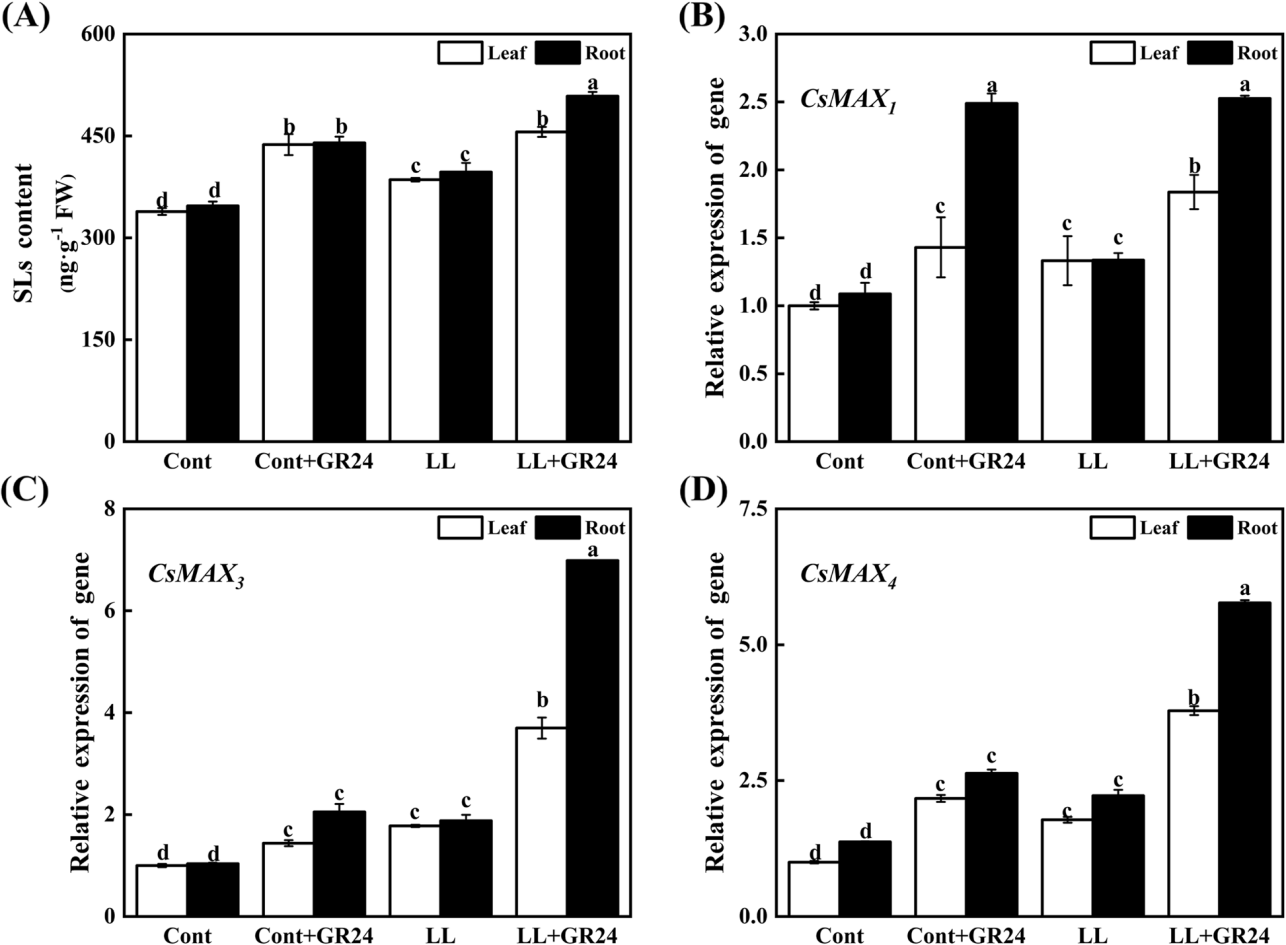
Effects of exogenous GR24 on the expression of strigolactone (**A**) and the strigolactone synthesis genes *CsMAX*_*1*_ (**B**), *CsMAX*_*3*_ (**C**) and *CsMAX*_*4*_ (**D**) in cucumber seedlings under low light stress. Note: The respective parameters were measured at 7 days after the start of low light stress and/or 10 μM GR24 treatments. Each histogram represents the mean value of three independent experiments, and the vertical bars indicate SEs (*n* = 3). Different letters indicate significant differences at *P* < 0.05, according to Duncan’s multiple range tests. Here, SLs, strigolactone; *MAX*_*1*_, more axillary growth 1; *MAX*_*3*_, more axillary growth 3; *MAX*_*4*_, more axillary growth 4. Cont, 0 μM GR24 + 500 μmolm^− 2^ s^− 1^ PPFD; Cont+GR24, 10 μM GR24 + 500 μmolm^− 2^ s^− 1^ PPFD; LL, 0 μM GR24 + 60 μmolm^− 2^ s^− 1^ PPFD; LL + GR24, 10 μM GR24 + 60 μmolm^− 2^ s^− 1^ PPFD

As shown in Fig. 6A, the strigolactone content in the roots and leaves of the cucumber treated with GR24 alone was 1.28- and 1.36-fold that of the untreated cucumber, respectively. Compared with the control, the strigolactone content in the roots and leaves of cucumber seedlings increased by 8.13 and 15.48% under low light stress, respectively. However, under low light stress, the content of strigolactone in cucumber roots treated with GR24 increased significantly, by 31.46% compared with the low light-only treatments. (Fig. 6A).

In the roots and leaves of cucumber seedlings under GR24 under low light stress, strigolactone synthesis and the transcriptional abundance of signal transduction genes, namely, *MAX*_*1*_*, MAX*_*3*_*, MAX*_*4*_ and *MAX*_*2*_, were significantly upregulated (Figs. 5 and 6B-D). In cucumber seedlings that were not treated with GR24, the expression of strigolactone synthesis genes in roots was slightly higher than that in leaves, but the differences were not significant. However, in cucumber seedlings under the combined treatment of low light stress and GR24, the expression of the strigolactone synthesis genes *MAX*_*1*_*, MAX*_*3*_ and *MAX*_*4*_ in roots was increased by 37.51, 88.89 and 52.34%, respectively, compared with the expression in leaves. Compared with low light stress alone, the expression levels of the *MAX*_*1*_*, MAX*_*2*_*, MAX*_*3*_ and *MAX*_*4*_ genes in leaves under the combined treatment of low light stress and GR24 were significantly increased by 37.91, 125.88, 108.04 and 112.75% on the 7th day. At the same time, the expression levels of the *MAX*_*1*_*, MAX*_*2*_*, MAX*_*3*_ and *MAX*_*4*_ genes in cucumber roots were significantly increased by 89.07, 159.23, 271.85 and 156.63%, respectively, which implied that exogenous GR24 application might participate in endogenous strigolactone induction and regulation of strigolactone biosynthesis and signal transduction to mitigate low light-induced damage to cucumber seedlings.

### Effects of GR24 on oxidative damage and *RBOH* gene expression under low light stress

**Fig. 7:**
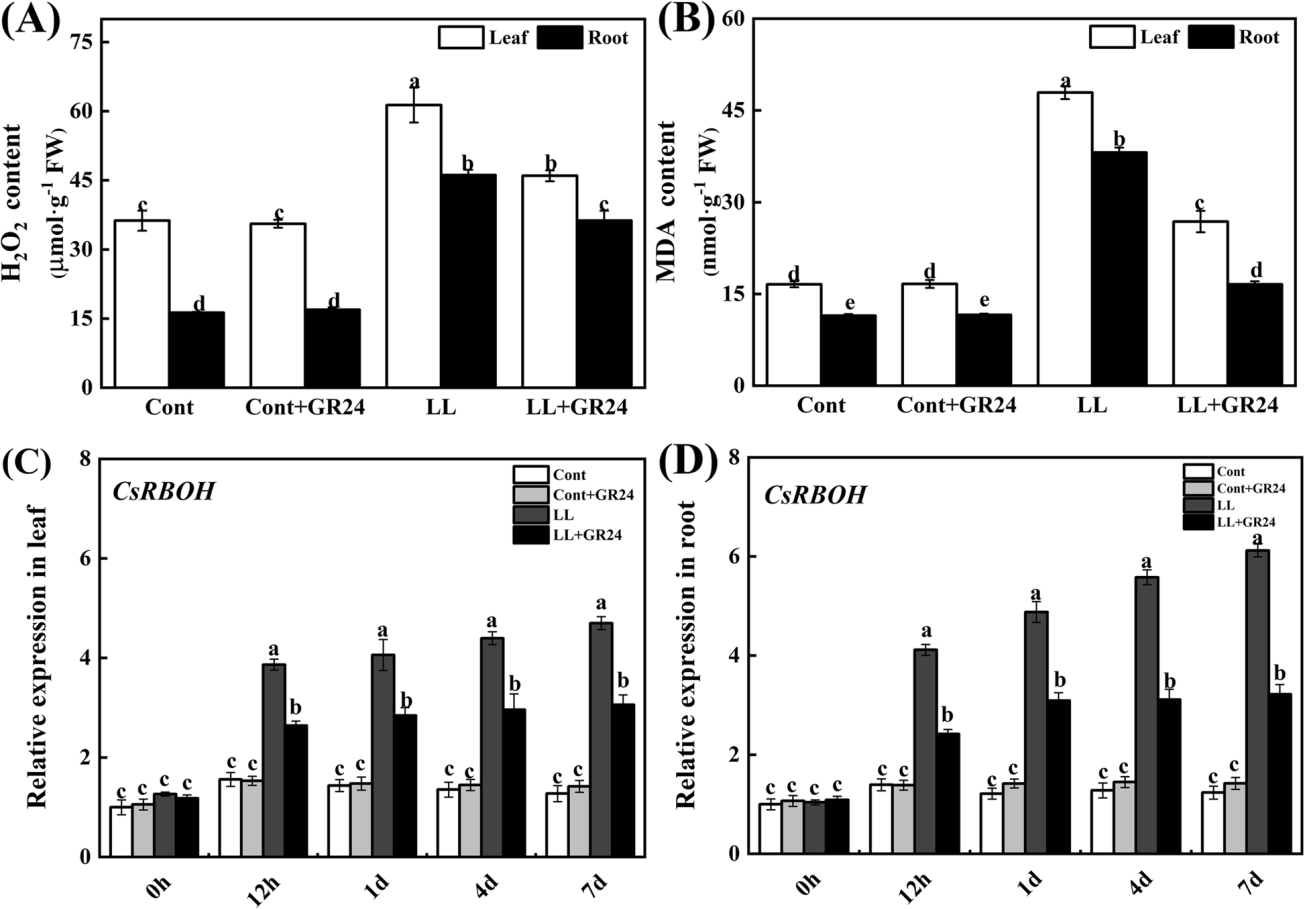
Effects of exogenous GR24 on H2O2 (**A**), MDA (**B**) and *RBOH* gene expression in cucumber seedling roots (**C**) and leaves (**D**) under low light stress. Note: The contents of H_2_O_2_ and MDA were measured at 7 days after the start of low light stress and/or 10 μM GR24 treatments. The samples used to determine gene expression were taken at 0 h, 12 h, 1 d, 4 d and 7 d after the start of low light stress and/or 10 μM GR24 treatments. Each histogram represents the mean value of three independent experiments, and the vertical bars indicate SEs (*n* = 3). Different letters indicate significant differences at *P* < 0.05, according to Duncan’s multiple range tests. Cont, 0 μM GR24 + 500 μmolm^− 2^ s^− 1^ PPFD; Cont+GR24, 10 μM GR24 + 500 μmolm^− 2^ s^− 1^ PPFD; LL, 0 μM GR24 + 60 μmolm^− 2^ s^− 1^ PPFD; LL + GR24, 10 μM GR24 + 60 μmolm^− 2^ s^− 1^ PPFD. Here, *RBOH*, respiratory burst oxidase homologue

As shown in Fig. 7A and B, after GR24 was used alone, the H_2_O_2_ and MDA contents in cucumber leaves and roots did not change significantly. However, compared with the control, the contents of H_2_O_2_ and MDA in cucumber leaves/roots significantly increased by 69.12/183.60% and 189.04/232.34% under low light stress, respectively. Strikingly, GR24 supplementation under low light stress significantly reduced the H_2_O_2_ and MDA contents in leaves/roots by 25.00/21.39% and 44.00/56.50%, respectively, compared with those under low light stress. It has been widely demonstrated that the *RBOH* gene encoding an ROS-forming enzyme is induced under stress conditions, and the relative expression of *RBOH* in the leaves and roots was markedly elevated throughout the low light stress duration (Fig. 7C and D), reaching approximately 6.11- and 4.69-fold from the initial time to 7 d of stress treatment, respectively. In contrast, GR24-treated seedlings showed downregulation of the expression of the same genes in the leaves and roots compared to that in low light-stressed seedlings, and the expression was reduced by 47.43 and 34.90% at 7 days of stress, respectively.

### Effects of GR24 on the antioxidant enzyme activity of cucumber seedlings under low light stress

**Fig. 8:**
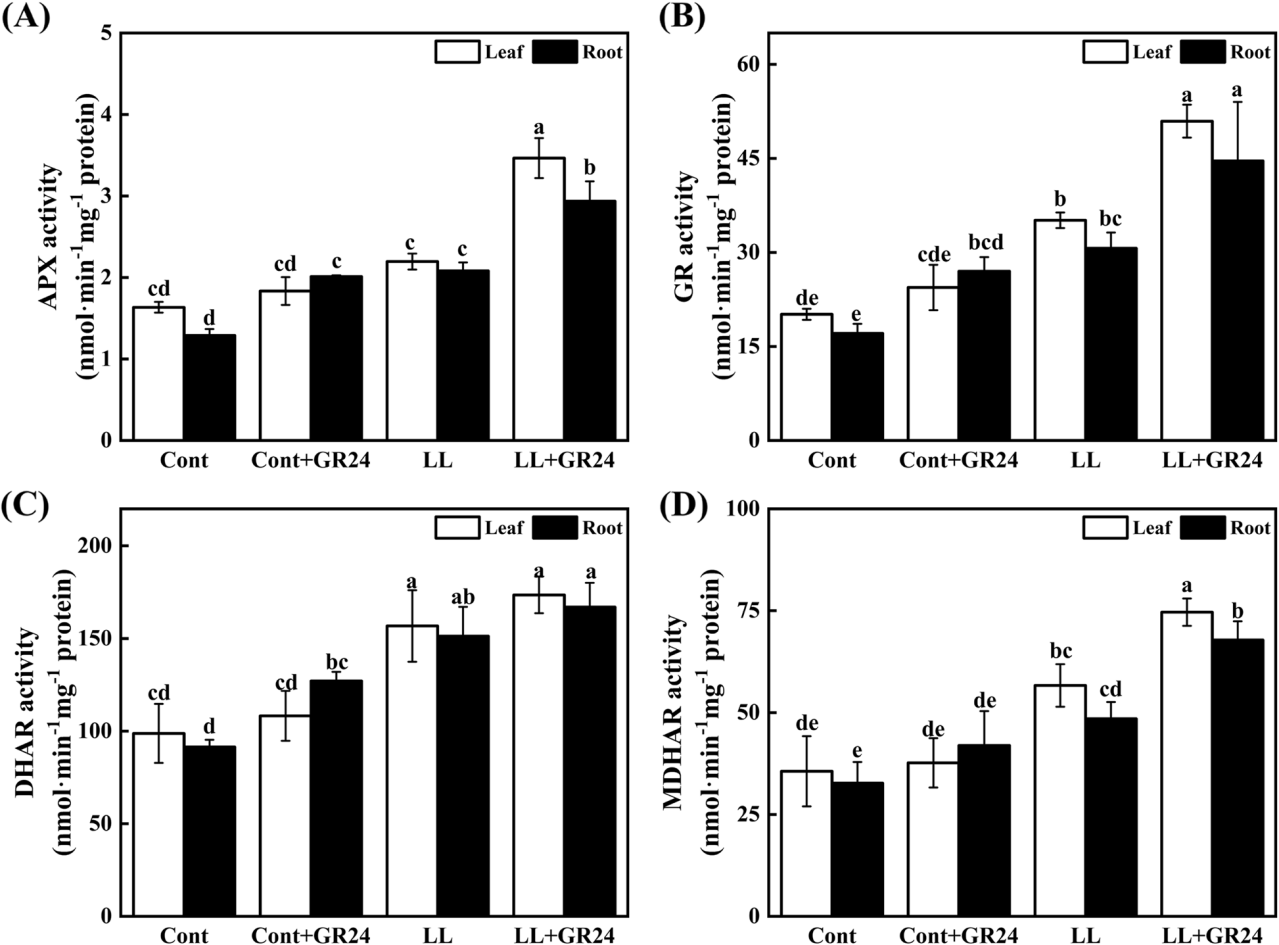
Effects of exogenous GR24 on the enzyme activities of APX (**A**), GR (**B**), DHAR (**C**) and MDHAR (**D**) in cucumber seedlings under low light stress. Note: The respective parameters were measured at 7 days after the start of low light stress and/or 10 μM GR24 treatments. Each histogram represents the mean value of three independent experiments, and the vertical bars indicate SEs (*n* = 3). Different letters indicate significant differences at *P* < 0.05, according to Duncan’s multiple range tests. Cont, 0 μM GR24 + 500 μmolm^− 2^ s^− 1^ PPFD; Cont+GR24, 10 μM GR24 + 500 μmolm^− 2^ s^− 1^ PPFD; LL, 0 μM GR24 + 60 μmolm^− 2^ s^− 1^ PPFD; LL + GR24, 10 μM GR24 + 60 μmolm^− 2^ s^− 1^ PPFD. Here, APX, ascorbate peroxidase; GR, glutathione reductase; DHAR, dehydroascorbate reductase; MDHAR, monnodehydroascorbate

To study the role of strigolactone in alleviating low light stress through antioxidant activity, we analysed the transcriptional abundance of *APX, GR, DHAR* and *MDHAR* in the leaves and roots of cucumber seedlings (Fig. 5) and the antioxidant enzyme activity (Fig. 8). Low light stress significantly stimulated the transcriptional abundance of the *APX, GR, DHAR* and *MDHAR* genes compared with the control, while the expression levels of these genes were further positively modulated in GR24-treated seedlings under low light stress (Fig. 5). Compared with the control conditions, the activities of APX, GR, DHAR and MDHAR in leaves/roots under low light stress increased by 34.34/61.37%, 74.49/79.49%, 58.73/65.48% and 59.20/48.34%, respectively. Importantly, treatment with GR24 under low light stress markedly increased the activities of APX, GR, DHAR and MDHAR compared with those under low light stress alone by 88.98/45.97%, 108.70/65.29%, 60.37/31.35% and 98.15/61.66%, respectively. In contrast, the GR24 treatment under low light stress increased the activities of APX, GR, DHAR and MDHAR in leaves/roots by 57.84/41.04%, 45.03/45.46, 10.70/10.35% and 31.73/39.84%, respectively, compared with those under low light stress. In addition, under only GR24 treatment, the activities of APX and GR were increased by 12.20/55.92% and 21.26/57.95% in leaves/roots compared to the control levels, respectively.

### Effects of GR24 on the antioxidant contents of cucumber seedlings under low light stress

**Fig. 9:**
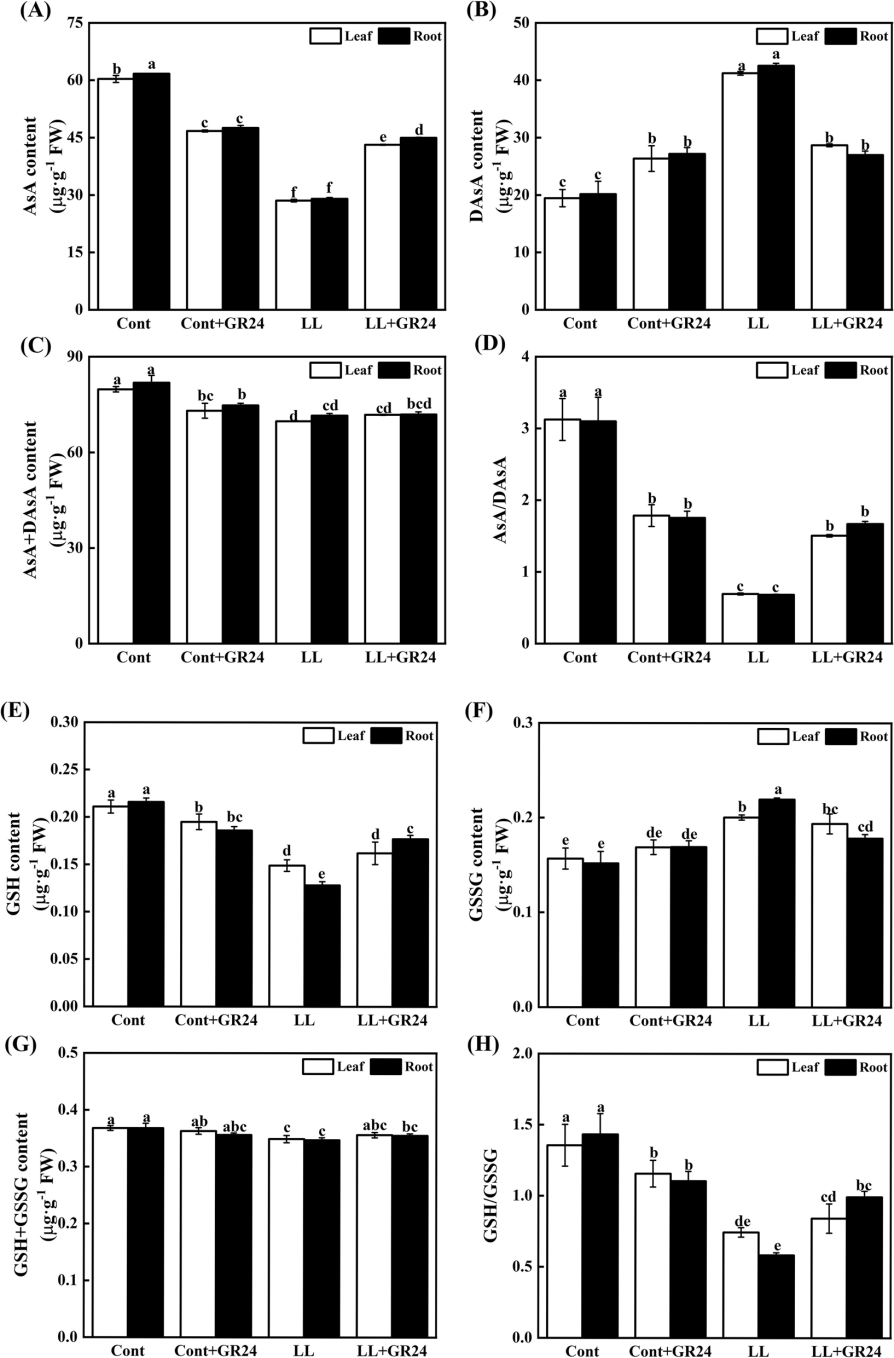
Effects of GR24 treatment on AsA(**A**), DAsA(**B**), and AsA + DAsA contents (**C**), the AsA/DAsA ratio (**D**), GSH(**E**), GSSG(**F**), and GSH + GSSG(**G**) contents and the GSH/GSSG ratio (**H**) in cucumber seedlings under low light stress. Note: The respective parameters were measured at 7 days after the start of low light stress and/or 10 μM GR24 treatments. Each histogram represents the mean value of three independent experiments, and the vertical bars indicate SEs (*n* = 3). Different letters indicate significant differences at *P* < 0.05, according to Duncan’s multiple range tests. Cont, 0 μM GR24 + 500 μmolm^− 2^ s^− 1^ PPFD; Cont+GR24, 10 μM GR24 + 500 μmolm^− 2^ s^− 1^ PPFD; LL, 0 μM GR24 + 60 μmolm^− 2^ s^− 1^ PPFD; LL + GR24, 10 μM GR24 + 60 μmolm^− 2^ s^− 1^ PPFD. Here, AsA, reduced ascorbate; DAsA, oxidized ascorbate; GSH, reduced glutathione; GSSG, oxidized glutathione

Compared with those in control seedlings, treatment with GR24 only reduced the contents of ASA, AsA + DAsA, GSH and GSH + GSSG in leaves/roots by 22.58/22.96%, 8.44/8.73%, 7.72/13.58% and 1.15/3.27% (Fig. 9) but increased the contents of DAsA and GSSG in leaves/roots by 35.46/34.84% and 7.68/11.35%, thereby reducing the values of AsA/DAsA and GSH/GSSG in leaves/roots by 42.85/43.43% and 14.66/22.86%, respectively. Low light stress significantly reduced the AsA and GSH contents in leaves by 52.70 and 29.32%, respectively, and in roots by 53.01 and 40.73%, respectively. In contrast, compared with those in the control seedlings, the DAsA contents increased by 111.89 and 111.07% in leaves and roots, respectively, while the GSSG contents increased by 27.81 and 44.45%, respectively. Compared with low light stress alone, the application of GR24 under low light stress increased the levels of ASA, AsA + DAsA, GSH and GSH + GSSG in leaves/roots by 51.07/54.97%, 2.90/0.52%, 8.74/38.18% and 1.82/2.20% but reduced the contents of DAsA and GSSG in leaves/roots by 30.46/36.60% and 3.33/18.76%, thereby increasing the values of AsA/DAsA and GSH/GSSG in leaves/roots by 117.24/144.58% and 12.45/70.08%, respectively.

## Discussion

As a hormone, strigolactone plays a key role in regulating plant growth and development and photosynthesis and responding to various abiotic stresses [13, 18, 34, 35].. Bu et al. have shown that *AtMAX*_*2*_ in the strigolactone signalling pathway is involved in the regulation of abiotic stress in plants, and this regulation process may depend on ABA [19]. At the same time, strigolactone can also affect the production of H_2_O_2_ and NO, and S-type anion channel *SLAC1* activity regulates the opening and closing of stomata, and this process does not depend on the ABA pathway [20]. The interplay of strigolactone and ABA regulates changes in the concentration and signalling of strigolactone during salinity stress [36]. Exogenous GR24 is a synthetic strigolactone analogue that can promote high strigolactone concentrations in plants [23]. Studies have revealed that *MAX*_*1,*_ *MAX*_*3*_ *and MAX*_*4*_ gene*-*encoded enzymes are the key substances of the strigolactone synthesis pathway, and the content of endogenous strigolactone in the mutant is reduced, while the *MAX*_*2*_ gene-encoded leucine-rich repeat sequence F-box protein can specifically recognize the substrate to promote the signal transduction of strigolactone. Plants of several species that are deficient in strigolactone biosynthesis or signalling-related genes show decreased stress tolerance, while application of a synthetic strigolactone analogue increases tolerance to drought stress in Arabidopsis and wheat [18, 22, 25, 37–42]. In this study, in the roots and leaves of cucumber seedlings under low light stress or GR24 treatment, the transcriptional abundance of strigolactone synthesis and signal transduction genes *MAX*_*1*_*, MAX*_*3,*_ *MAX*_*4*_ *and MAX*_*2*_ was slightly upregulated, which resulted in the endogenous strigolactone content being slightly increased. Under the cotreatment of low light stress and GR24, the above indicators all increased significantly, especially in roots. (Figs. 5 and 6).

In general, light directly affects the photosynthesis and photomorphogenesis of plants. The former provides necessary energy for the formation of chlorophyll and biomass; the latter controls the growth and development of plants through light signals. The change in plant biomass is a comprehensive manifestation of the response to adverse stress [4]. From the results of this article, it is obvious that cucumber plants experienced growth and development obstacles after low light treatment, such as plant growth inhibition and reduced biomass production, while GR24 treatment alleviated the symptoms of low light stress (Fig. 1, Table 2), which indicates that the application of GR24 can maintain better photosynthesis and nutrient absorption and metabolism levels of tomato plants under low light stress. Cell organelles, such as chloroplasts, are the site for most photosynthetic processes and are also affected by low light stress [4, 7]. The chlorophyll content is used as an indicator of chloroplast development and photosynthetic performance. In this work, GR24 significantly increased the contents of chlorophyll *a* and total chlorophyll *a + b* under low light conditions. In addition, GR24 also increased the chlorophyll a/b ratio because GR24 induced an increase in chlorophyll *a* and a reduction in chlorophyll *b*, which may have been related to the inhibition of chlorophyll-degrading enzyme activity by regulating chlorophyllase (Table 3). In addition, GR24 may regulate the binding of chlorophyll to membrane proteins to maintain the stability of the chloroplast thylakoid membrane and enhance photosynthetic capacity [4, 43, 44]. Therefore, regulating chlorophyll components and photosynthesis may be another strategy by which GR24 helps plants adapt to low light stress.

Photosynthesis is the basis of crop yield and crop quality. Decreases in photosynthetic capacity are usually mainly due to stomatal and/or nonstomatal limitations. The observation of key parameters with chlorophyll fluorescence imaging can directly reflect the photochemical efficiency of plants, which is the key to our understanding of photosynthetic physiology [27, 28, 31]. In this study, as the treatment time increased, the gas exchange parameters such as the net photosynthetic rate (Pn), stomatal conductance (Gs) and transpiration rate (Tr) of cucumber seedling leaves under low light stress were all significantly reduced. However, exogenous GR24 significantly reduced the negative effects caused by low light stress and improved related parameters (Fig. 2). Therefore, the reduction in Pn in plants treated with low light is mainly attributed to nonstomatal limitation, including limited carbon assimilation or poor performance of the photosynthetic apparatus, while GR24 improves photosynthetic efficiency by reducing nonstomatal limitation [45]. In addition, the values of Fv/Fm, ΦPSII, ΦNPQ, qP and NPQ were significantly reduced under low light stress. With the supplementation of GR24, the above parameters were improved to a certain extent (Fig. 3). These results indicate that GR24 may restore photosynthetic processes by maintaining the stability of the PSII supercomplex, enhancing the turnover of the D1 protein, improving photosynthetic electron transport and the demand for ATP and NADPH in the Calvin cycle, improving the ability of heat dissipation pathways and reducing the excitation pressure of the PSII reaction centre to reduce the degree of photoinhibition [43] and damage to the photosynthetic capacity of cucumber plants under low light conditions.

The photochemical efficiency of PSII can directly affect the CO_2_ assimilation ability of higher plants. The initial product of CO_2_ fixation by chloroplasts is triose phosphate, which is transformed into sucrose after being transported to the cytoplasm. Sucrose is an important form of photosynthetic product for transport. Sucrose can be used not only as a source of carbon and energy in plants but also as a signalling substance to regulate tissue growth and sugar-mediated feedback inhibition of photosynthesis. Sucrose metabolism plays a key role in development, stress response, and yield formation, primarily through producing a series of varied sugars to promote growth and synthesize necessary chemical compounds in plants. Sucrose synthase (SS) and sucrose phosphate synthase (SPS) are the key enzymes in the sucrose synthesis pathway. In leaves, SPS plays a role as the limiting factor in the export of photoassimilates to sink tissues [29, 31, 46–49]. In this study, *SPS* and *SS* gene expression and sucrose synthase and sucrose phosphate synthase enzyme activity were inhibited under low light stress, so the sucrose and soluble sugar contents in cucumber leaves were significantly reduced. With GR24 supplementation, the expression of these genes and the activities and contents of the enzymes that encode both were significantly increased (Figs. 4 and 5).

The decrease in photochemical efficiency is also related to membrane lipid peroxidation. As a toxic substance, ROS act as a signalling molecule to regulate abiotic stress effects in plants. Abiotic stress can disturb homeostasis in the cell and rapidly induce the production of a large amount of ROS through aerobic metabolism, thereby triggering oxidative stress, membrane lipid peroxidation and metabolic disorders. To maintain the redox balance, plants usually quench ROS with the help of antioxidant enzymes and nonenzymatic systems. Antioxidant enzymes and other antioxidants are important components to eliminate ROS under abiotic stress [31, 33]. Some recent studies have found that several stresses can stimulate the *RBOH* gene to induce the production of ROS, which is essential for rapid defence signalling in the early stage of stress [30, 33].. In this study, low light stress induced an increase in *RBOH*, *APX, GR, DHAR* and *MDHAR* expression and antioxidant enzyme activity and catalysed the production of H_2_O_2_ and MDA. In addition, low light stress also reduced the contents of AsA and GSH in cucumber seedlings. After GR24 application under low light stress, the expression levels of these genes were further positively modulated, and the contents of AsA + DAsA, GSH and GSH + GSSG were all increased, while the contents of DAsA and GSSG decreased, thereby increasing the values of AsA/DAsA and GSH/GSSG. These factors all improve the ability of cucumber to remove H_2_O_2_, thereby alleviating the accumulation of MDA.

## Conclusion

**Fig. 10:**
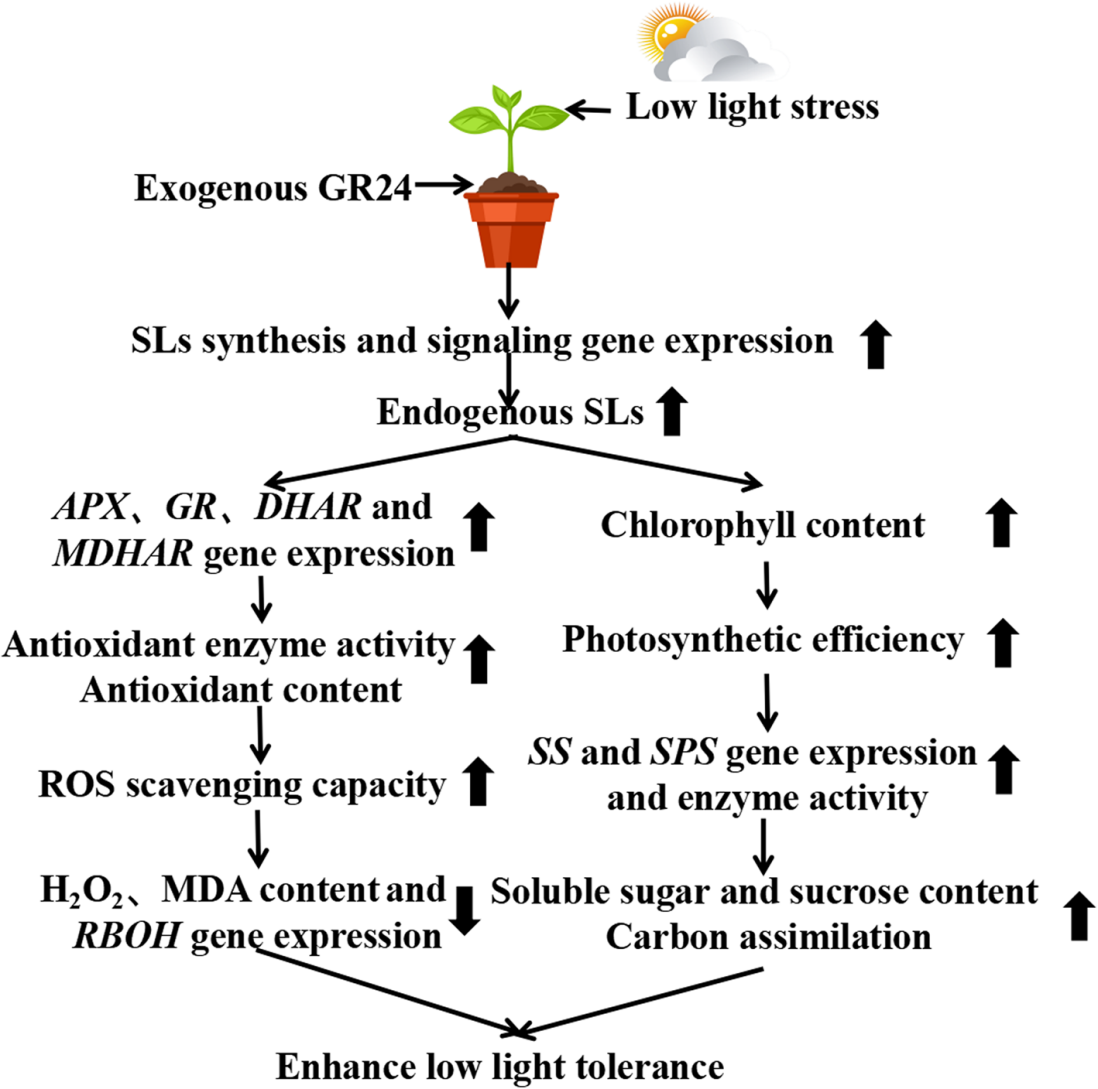
A proposed model showing how strigolactone enhances low light tolerance in cucumber seedlings

In summary, as shown in Fig. 10, the application of GR24 significantly alleviated the growth inhibition of cucumber seedlings induced by low light stress. This may have been due to exogenous GR24 inducing the synthesis of endogenous strigolactone, which regulates the key enzyme activity of antioxidant metabolism, and its gene expression reduces oxidative damage under low light stress. In addition, GR24 improved the photosynthetic capacity of cucumber seedlings under low light stress, which increased the accumulation of biomass and enhanced cucumber resistance to low light stress.

## Data Availability

The datasets generated and analysed during the current study are available from the corresponding author on reasonable request. All sequences generated by sequencing for this study are available in the Cucumber Genome Database (http://cucurbitgenomics.org/organism/2).

## Abbreviations

ABA: Abscisic acid
APX: Ascorbate peroxidase
AsA: Ascorbate
CAT: Catalase
Chl: Chlorophyll
Ci: Intercellular CO_2_ concentration
DAsA: Oxidized ascorbate
DHAR: Dehydroascorbate
EC: Electrical conductivity
ELISA: Enzyme-linked immunosorbent assay
Fm: Maximum fluorescence yield
Fm′: Fluorescence maximum in the light
F0: Minimum fluorescence yield
Fs: Steady-state fluorescence yield
Fv/Fm: Maximum quantum yield of PSII
GR: Glutathione reductase
Gs: Stomatal conductance
GSSG: Oxidized glutathione
GSH: Glutathione
H_2_O_2_: Hydrogen peroxide
KI: Potassium iodine
MAX: More axillary growth
MDA: Malondialdehyde
MDHAR: Monodehydroascorbate reductase
NPQ: Non-photochemical quenching
PIF: Phytochrome interacting factor
Pn: Net photosynthetic rate
POD: Peroxidase
PPFD: Photosynthetic photon flux density
qL: Photoinhibitory quenching
qP: Photochemical quenching coefficient
RBOH: Respiratory burst oxidase
RNA: Ribonucleic acid
ROS: Reactive oxygen species
SL: Strigolactone
SOD: Superoxide dismutase
SPL: Squamosa promoter binding protein-like
SPS: Sucrose phosphate synthase
SS: Sucrose synthase
TCA: Trichloroacetic acid
TMB: Tetramethylbenzidine
Tr: Transpiration rate
ФPSII: Effective photochemical quantum yield of PSII
ФNPQ: Regulatory energy dissipation quantum yield of PSII
ФNO: Nonregulatory energy dissipation quantum yield of PSII
